# Prediction of Antileishmanial Compounds: General Model, Preparation, and Evaluation of 2-Acylpyrrole Derivatives

**DOI:** 10.1021/acs.jcim.2c00731

**Published:** 2022-08-10

**Authors:** Carlos Santiago, Bernabé Ortega-Tenezaca, Iratxe Barbolla, Brenda Fundora-Ortiz, Sonia Arrasate, María Auxiliadora Dea-Ayuela, Humberto González-Díaz, Nuria Sotomayor, Esther Lete

**Affiliations:** † Departamento de Química Orgánica e Inorgánica, Facultad de Ciencia y Tecnología, Universidad del País Vasco / Euskal Herriko Unibertsitatea UPV/EHU, Apdo. 644, 48080 Bilbao, Spain; ‡ Department of Computer Science and Information Technologies, University of A Coruña (UDC), 15071, A Coruña, Spain; § BIOFISIKA. Basque Center for Biophysics CSIC-UPV/EHU, 48940, Bilbao, Spain; ∥ Departamento de Farmacia, Facultad de Ciencias de la Salud, Universidad CEU Cardenal Herrera, 46115 Alfara del Patriarca, Valencia, Spain; ⊥ IKERBASQUE, Basque Foundation for Science, 48011 Bilbao, Spain

## Abstract

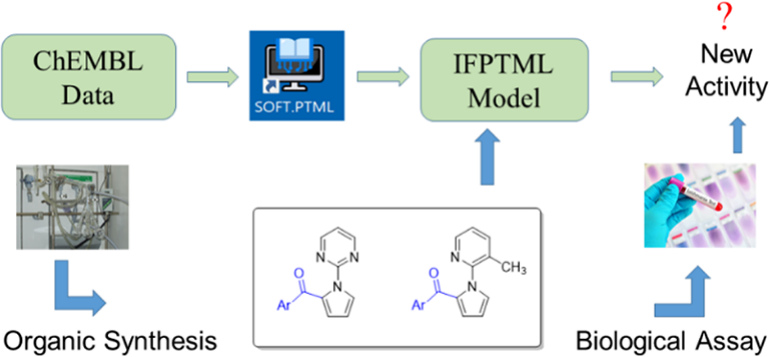

In this work, the SOFT.PTML tool has been used to pre-process a ChEMBL dataset of pre-clinical assays of antileishmanial compound candidates. A comparative study of different ML algorithms, such as logistic regression (LOGR), support vector machine (SVM), and random forests (RF), has shown that the IFPTML-LOGR model presents excellent values of specificity and sensitivity (81–98%) in training and validation series. The use of this software has been illustrated with a practical case study focused on a series of 28 derivatives of 2-acylpyrroles **5a**,**b**, obtained through a Pd(II)-catalyzed C–H radical acylation of pyrroles. Their *in vitro* leishmanicidal activity against visceral (*L. donovani*) and cutaneous (*L. amazonensis*) leishmaniasis was evaluated finding that compounds **5bc** (IC_50_ = 30.87 μM, SI > 10.17) and **5bd** (IC_50_ = 16.87 μM, SI > 10.67) were approximately 6-fold more selective than the drug of reference (miltefosine) in *in vitro* assays against *L. amazonensis* promastigotes. In addition, most of the compounds showed low cytotoxicity, CC_50_ > 100 μg/mL in J774 cells. Interestingly, the IFPMTL-LOGR model predicts correctly the relative biological activity of these series of acylpyrroles. A computational high-throughput screening (cHTS) study of 2-acylpyrroles **5a**,**b** has been performed calculating >20,700 activity scores *vs* a large space of 647 assays involving multiple *Leishmania* species, cell lines, and potential target proteins. Overall, the study demonstrates that the SOFT.PTML all-in-one strategy is useful to obtain IFPTML models in a friendly interface making the work easier and faster than before. The present work also points to 2-acylpyrroles as new lead compounds worthy of further optimization as antileishmanial hits.

## 1 Introduction

**Figure 1:**
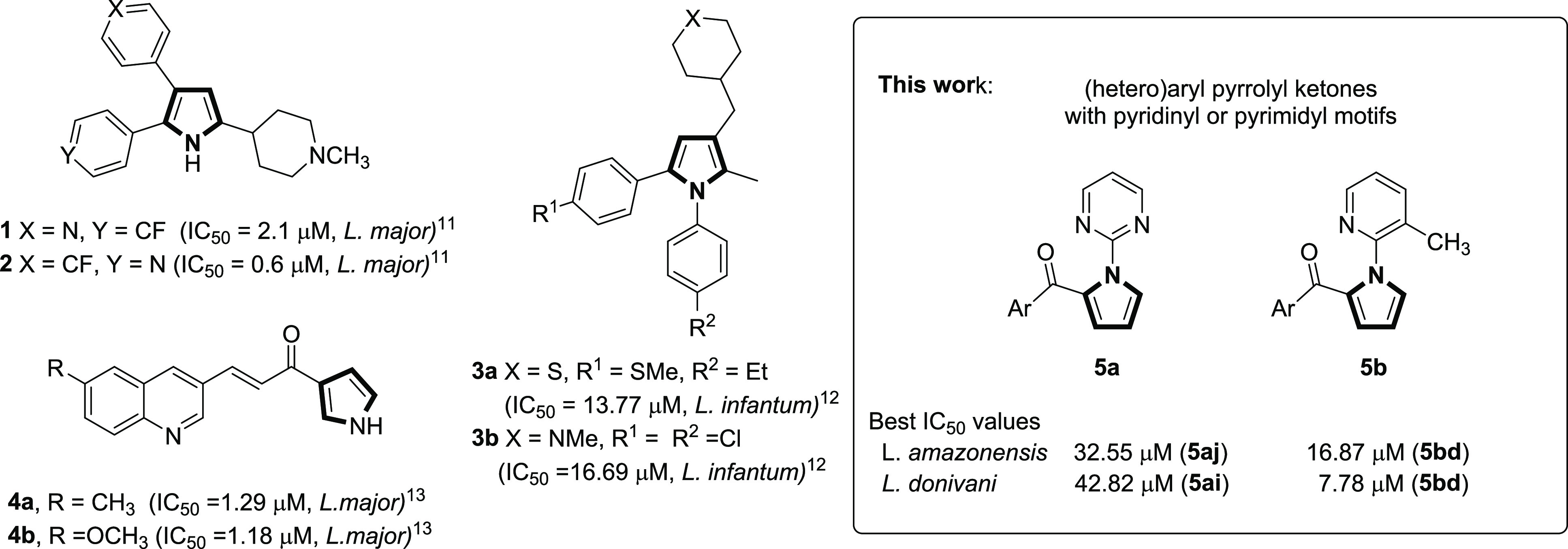
Antileishmanial activity of some synthetic compounds with the pyrrole motif (IC_50_ values are relative to in vitro assays on promastigotes for all compounds, except for **3**, where the values are relative to amastigotes).

Leishmaniasis is a parasitic disease, caused by *Leishmania* genus protozoan pathogens, that may present different clinical manifestations including cutaneous (CL), visceral or kala-azar (VL), post-kala-azar dermal leishmaniasis (PKDL), and mucocutaneous (MCL) leishmaniasis. As all neglected diseases, leishmaniasis remains a major global health problem as it is endemic in around 100 countries with more than 350 million people at risk.^1^ Treatment of leishmaniasis relies mainly in a few drugs: pentavalent antimonials (ampB), paromomycin, pentamidine, liposomal amphotericin B, fluconazole, and miltefosine, depending on the etiological species, the infection type, and also the geographical region because of the increasing number of resistant strains. Additionally, the use of these drugs is associated with a number of severe side effects related to their toxicity.^2−5^ Therefore, it is necessary to identify new effective antileishmanial compounds with chemotypes other than the ones in clinical use. In this context, nitrogen heterocycles can be considered privileged scaffolds because approximately 60% of U.S. FDA approved small-molecule drugs contain a nitrogen heterocycle.^6^ In particular, the pyrrole core has attracted our attention because this motif is embedded in a variety of natural products (e.g., prodiginines,^7^ bromopyrrole,^8^ and spiroindimicin alkaloids^9^) with antiparasitic activity.^10^ Regarding synthetic derivatives, pyridinyl aryl pyrroles **1** and **2** have proven to be inhibitors of casein kinase 1 that block the growth of *Leishmania major* promastigotes *in vitro.*(11) 1,2-Diarylpyrroles **3** have been identified as a new class of compounds active against the amastigote stay of *Leishmania infantum* by inhibiting the trypanothione reductase.^12^ On the other hand, 2-acylpyrrole derivatives **4** also exhibited promising antileishmanial profiles (Figure 1).^13^

Additionally, it has been reported that pyrrole-indolinone SU11652, a sunitinib analog, targets the nucleoside diphosphate kinase from *Leishmania* parasites.^14^ We recently synthesized 2-acylpyrroles through Pd(II)-catalyzed radical C–H acylation of pyrrole derivatives.^15^ This efficient and flexible protocol allowed us to collect a small library of 2-acylpyrroles **5**, variably substituted on the aryl ring, and with a pyrimidine (series **5a**) or pyridine (series **5b**) ring linked to the nitrogen atom of the pyrrole nucleus (Figure 1). These structural features make our pyrrole derivatives interesting candidates to be tested as potential antileishmanial compounds.

In this context, cheminformatic modeling can be a good option to reduce the development cost and increase the probability of finding new antileishmanial hits. Classic cheminformatic models focus on accelerating the antiparasitic drug discovery process by reducing the number of compounds to be assayed by trial-and-error tests. However, in addition to the large number of compounds to be tested, other factors may play a role slowing down this process. For example, the large number of combinations of biological parameters (MIC, IC_50_, pK_i_, *etc*.), parasite species, parasite stages, or target proteins greatly increases the time and cost *per* compound to be tested. Unfortunately, classic cheminformatic models fail to perform multiobjective optimization of antiparasitic compounds due to the difficulty of encoding multiple boundary conditions (parameter, protein, cell line, species, parasite stage, *etc*.) of assay and the need to obtain this information from many different data sources. We have recently reported the first PTML [(perturbation theory (PT) + machine learning (ML)] model that is capable of both explaining a very large dataset of preclinical assays of antileishmanial compounds and predicting the activity of new heterocycles (e.g., two series of pyrroloisoquinolines synthesized by our group) against different species of *Leishmania*.^16^ Nevertheless, the development process of this first PTML model and its subsequent use for the prediction of new antileishmanial hits were laborious.

On the other hand, we have coined the term IFPTM [information fusion (IF) + perturbation theory (PT) + machine learning (ML)] for a new algorithm designed for multiobjective optimization of compounds. When the IF stage is missing, only the term PTML is used.^17,18^ These IFPTML models have been used in medicinal chemistry, proteomics, metabolomics, and nanotechnology.^19,20^

The first phase (IF + PT) of these IFPTML models consists of merging information from different sources and/or transforming the original variables into PT operators (PTOs). These PTOs are new input variables useful for encoding information about multiple assay conditions from different sources. For example, PTOs can be used to encode information about protein targets, cell lines, microbial metabolic networks of target organisms, nanoparticle carriers of the drug, *etc*.^19,20^ Next, the IFPTML workflow enters into the ML phase using classic ML algorithms. Until recently, training PTML models required running different software for each stage of the algorithm (IF, PT, and ML),^19,20^ as was the case for our previous IFPTML model for antileishmanial compounds.^16^ A calculation sheet was needed to run the first phase, ML software to seek the model, and a new calculation sheet to run predictions. This problem drew the attention of cheminformatics software developers to the need for new platforms to unify the different steps of IFPMTL analysis. To this end, we have introduced the QSAR-Co tool that jointly runs the PT and ML stages of the algorithm.^21^ However, QSAR-Co cannot run IF procedures to calculate multilabel PTOs or reference functions that encode multiple assay conditions at the same time. Furthermore, QSAR-Co only calculates one class of PT operators, called single-condition moving averages. Consequently, it needs as many PTOs as boundary conditions are present in the problem, which implies a significantly higher number of variables to explore with respect to the multilabel PTOs used in IFPTML algorithms.^19,20^ These PTOs have proven to be very useful in reducing the problem dimensionality, as in the case of ChEMBL antileishmanial pre-clinical assays dataset.^16^

Therefore, we introduced the SOFT.PTML studio tool, which has the possibility of calculating multilabel PTOs, including multilabel/multicondition reference functions, moving averages, co-variances, *etc*. SOFT.PTML has been used successfully in nanotechnology and medicinal chemistry.^22,23^ In the present work, we report for the first time the use of SOFT.PTML to seek IFPTML models for antileishmanial compounds, performing a comparative study of different ML algorithms. We have also carried out a predictive study of a series of 2-acylpyrroles, previously synthesized by our group,^15^ together with experimental preparation of new samples for assay and their *in vitro* leishmanicidal testing. Some of the 2-acylpyrroles tested compare favorably with respect to miltefosine (reference compound) in terms of activity and toxicity. This work opens a new experimental line of research focused on the synthesis and optimization of antileishmanial compounds as 2-acylpyrrole derivatives. It also lays the ground for the development of faster and user-friendlier IFPTML models for other neglected tropical diseases.

**Figure 2:**
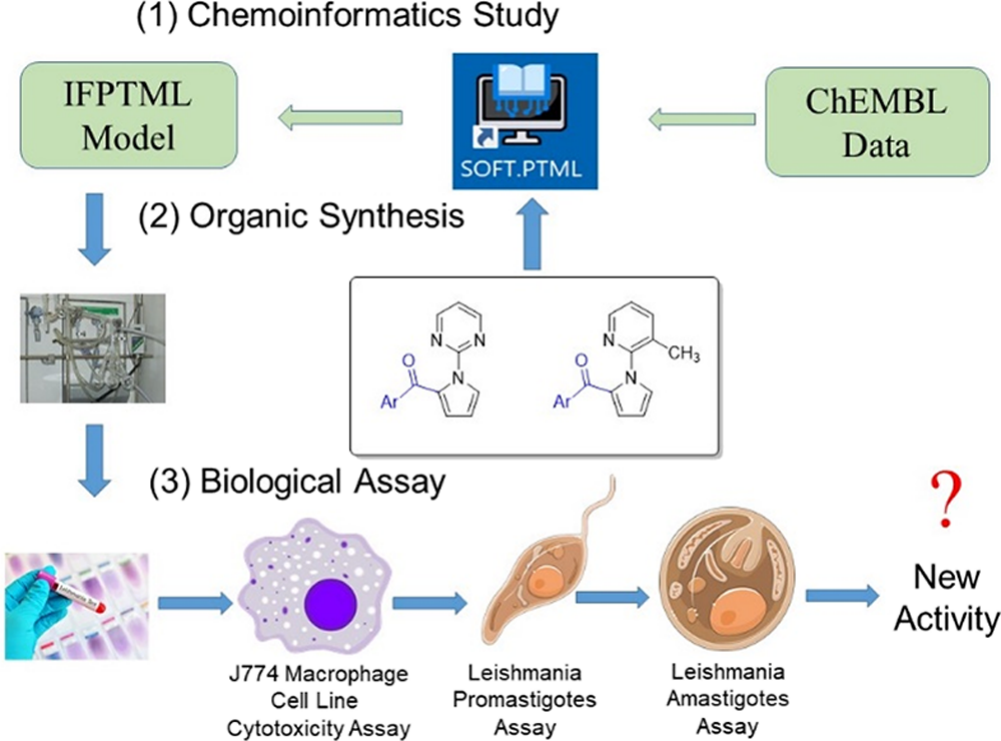
General workflow.

The general flowchart showing the interconnections between the different parts of this work: (1) chemoinformatics study, (2) organic synthesis, and (3) biological assays, is depicted in Figure 2.

## 2 Materials and Methods

### 2.1 Computational Methods

#### 2.1.1 IFPTML Model Basis

An IFPTML model is proposed to calculate the values of the antileishmanial biological activity scoring function f(*v_ij_*)_calc_ of the *i*th query compound in the *j*th assay with multiple boundary conditions **c**_j_ = [c_0_, c_1_, c_2_, c_jmax_]. In these classification models, the f(*v_ij_*)_calc_ function, which gets dimensionless values, is used to score the propensity of the *i*th compound to reach a certain level of the biological activity values v_ij_ (see next section).^24^ Consequently, the values of f(*v_ij_*)_calc_ can be used directly to compare the relative propensity of two different compounds to reach a certain level of biological activity in the *j*th assay compared to a threshold value cutoff_j_. They can also be used to compare the behavior of the same compound in two different assays. The IFPTML model uses two types of input variables [functions of reference f(*v_ij_*)_ref_ and perturbation theory operators PTO_k_(**c**_j_)] to calculate the f(*v_ij_*)_calc_ output values. Thus, the IFPTML model starts with the values of a function of reference f(*v_ij_*)_ref_, which are used to characterize/identify the kind of biological activity to be modeled. Next, the values of the PTO_k_(**c**_j_) functions are used to measure the effect of perturbations over the biological activity outcome. PTO_k_(**c**_j_) functions quantify perturbations/deviations in the structure of the *i*th compound and/or in the conditions assay **c**_j_ compared to a set of reference compounds.^24^ In the next section, the pre-processing of the raw data to construct the f(*v_ij_*)_ref_ and PTOk(**c**_j_) functions is explained. IFPTML linear models have the following form (eq 1):
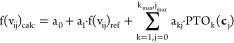
1

**Figure 3:**
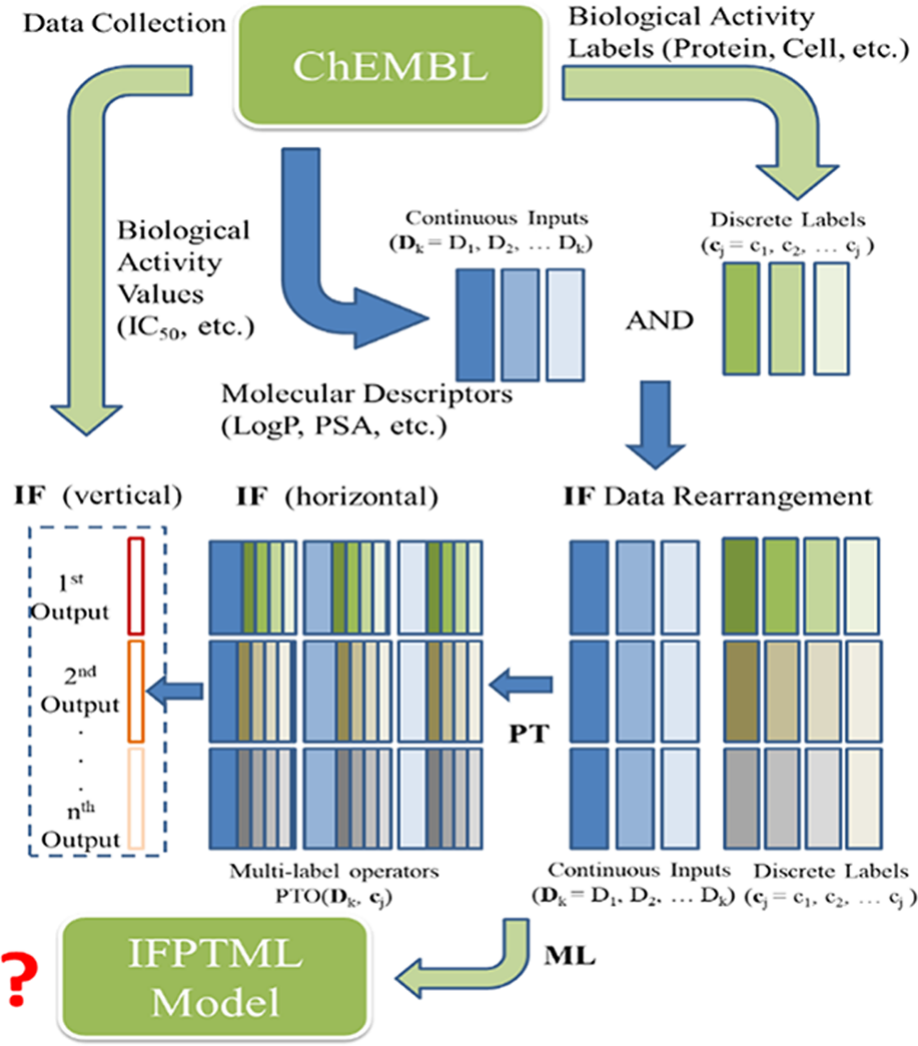
IFPTML method workflow illustrating the different phases (IF + PT + ML).

Figure 3 shows a workflow illustrating the integration of the different phases (IF + PT + ML) of the IFPTML analysis. First, the IF phase, which includes data collection, data rearrangement, and data fusion (horizontal and vertical), is run. The vertical IF involves aligning the output values v_ij_ for different output parameters (IC_50_, K_i_, *etc*.) in the same column. These parameters are then transformed into a Boolean variable f(*v_ij_*)_obs_ using different cutoff values, (see next section). Horizontal IF involves merging multiple labels from pre-clinical assays to form multicondition label variables **c**_j_ = [c_1_, c_2_, ... c_j_]. Next, the PT phase is run by calculating the PTO(D_k_, **c**_j_) operators that can encode structural information (D_k_) and multiple assay conditions (**c**_j_) at the same time. In the last phase, the different ML algorithms are run using the PTO(D_k_, **c**_j_) values as input. The following sections provide more details about the IFPTML steps.

#### 2.1.2 Data Pre-processing and IF Output (Horizontal IF)

The SOFT.PTML tool was used to pre-process a ChEMBL dataset of pre-clinical assays of antileishmanial compound candidates.^16^ This tool was also used to perform training/validation of alternative IFPTML models. These data included 145,851 biological activity values (v_ij_) for the *i*th compounds tested on the *j*th preclinical assays with boundary experimental conditions (labels) **c**_j_ = [c_0_, c_1_, c_2_, ... c_n_]. The values v_ij_ are expressed as different antileishmanial activity parameters with label c_0_, such as IC_50_, K_i_, EC_50_, *etc*. The SOFT.PTML discretization procedure was used in order to convert all the values of biological activity v_ij_ into a Boolean objective function f(*v_ij_*)_obs_ = 1 or 0. The function was implemented in the software is f(*v_ij_*)_obs_ = 1 IF (v_ij_ ≥ cutoff_j_ AND d(c_0_) = 1) OR (v_ij_ ≤ cutoff_j_ AND d(c_0_) = −1) ELSE f(*v_ij_*)_obs_ = 0. In this equation, ≥ means higher than (>) or equal to (=) the cutoff. By analogy, ≤ means lower than (<) or equal to (=) the cutoff. The different cutoff_j_ values are the cutoff values used for the different c_0_-labaled biological activity parameters (IC_50_, K_i_, EC_50_, *etc*.) in different *j*th assays. The desirability parameter d(c_0_) = 1 or – 1 had to be maximized or minimized to obtain an optimal biological effect. This IFPTML model is multioutput, i.e., it can predict multiple output probability of activity p(f(v_ij_) = 1) for the same *i*th drug entry. Consequently, to train the model, different values of the biological activity parameter v_ij_ (K_i_, IC_50_, etc.) have been transformed into Boolean variables f(v_ij_) = 1 OR 0. Since each biological activity parameter v_ij_ (K_i_, IC_50_, *etc*.) has a different scale and optimal region, different values of cutoff were used for each parameter v_ij_. These values of cutoff_j_ were determined by trying to balance both the optimal activity region and the number of cases in the two classes f(v_ij_) = 1 OR 0 for each parameter. The more representative values of cutoff_j_, desirability d(c_0_), and number of cases n(c_0_) in the dataset are listed in the Supporting Information 1 (CUTOFF sheet). The number of positive cases n(f(v_ij_) = 1) and resulting values of the reference function f(*v_ij_*)_ref_ = p(f(v_ij_) = 1) priori probability for different biological activity parameters c_0_ = property (units) were also listed.

#### 2.1.3 Input IF and Codification (Horizontal IF)

All the information on the boundary experimental conditions of the biological activity assays is codified by the vectors **c**_j_ = [c_0_, c_1_, c_2_, ...c_maxj_]. Members of this vector were regrouped, creating two partitions P(***c***_*j*_)_I_ = **c**_I_ and P(***c***_*j*_)_II_ = **c**_II_, which are also discrete variables. The value of the partition can be calculated by means of a horizontal IF process (see Figure 3) that consists on concatenating the text values of each label variable c_j_ included in the subset of variables I or II in the partition. That is, the first partition is calculated as P(***c***_*j*_)_I_ = concatenate (***c***_*j*_)_I_ = concatenate ([c_0_, c_1_, ... c_max_])_I_ = **c**_I_ and the second as P(***c**j*)_II_ = concatenate (***c**j*)_II_ = concatenate ([c_0_, c_1_, ... c_max_])_II_ = **c**_II_. These partitions independently encode information on the experimental conditions of preclinical assays P(***c***_*j*_)_I_ = **c**_I_ = Concatenate([c_0_, c_1_, c_2_, c_3_, c_4_, c_5_]) = c_0_ – c_1_ – c_2_ – c_3_ – c_4_ – c_5_ and on the nature and quality of data P(***c***_*j*_)_I_ = **c**_I_ = concatenate ([c_6_, c_7_, c_8_, c_9_, c_10_]).

On the other hand, the structural information is also expressed as a vector **D**_ki_ = [D_1i_, D_2i_, D_3i_, ...D_maxi_]. The elements of this vector are: D_1i_ = ALOGP_i_, the *n*-octanol/water partition coefficient; D_2i_ = PSA_i_, the topological polar surface area, and D_3i_ = NVLR, the number of violations to Lipinski’s rule of the structure of *i*th compound. The values of the molecular descriptors D_k_ were downloaded from ChEMBL and/or calculated with the software DRAGON^25^ for new compounds. Next, the IF process was carried out to calculate the multicondition PT operators PTO_k_(**D**_ki_, **c**_j_). Each PTO_k_(**D**_ki_, **c**_j_) variable is the expression of the fusion of structural information from one or multiple elements of **D**_i_ and one or multiple elements of **c**_j_. Therefore, a PTO_k_(**D**_ki_, **c**_j_) is a function or operator (PTO_k_) calculated to merge structural information **D**_i_ of the *i*th compound with the conditions **c**_i_ of the *j*th pre-clinical assay. The PTO_k_ can have many different forms and/or can be calculated for different subsets (domains) of the vectors **D**_ki_ and **c**_j_. First, the operators of type PTO(D_ki_, **c**_I_) = PTO(D_1i_, **c**_I_), PTO(D_2i_, **c**_I_), *etc*. or type PTO_k_(**D**_ki_, **c**_II_) = PTO(D_1i_, **c**_II_), PTO(D_2i_, **c**_II_), *etc*. are calculated. These calculated PTOs can take the form of multicondition moving average (MA) operators: ΔD_k_(**c**_I_) = D_ki_ – ⟨D_k_(**c**_I_)⟩ or ΔD_k_(**c**_I_) = D_ki_ – ⟨D_k_(**c**_II_)⟩. They measure the deviation (Δ) of the structure of the *i*th compound expressed by D_ki_ with respect to the average/expected values ⟨D_k_(**c**_j_)⟩ for all the compounds assayed under the same conditions **c**_I_ or **c**_II_.

#### 2.1.4 SOFT.PTML *In Silico* Screening of New Compounds

The new model was used to study a series of 28 di(hetero)aryl ketone derivatives (2-acylpyrroles) synthesized in our group.^16^ First, the SMILE codes of the 28 compounds were generated using ChemDraw Professional 20.1.^26^ Next, the values of the input variables D_1_ = LOGP, D_2_ = PSA, and D_3_ = NVLR were calculated. Then, these values were substituted on the SOFT.PTML model in order to obtain the probabilities of activity for each compound in different biological assays. A simulation of the biological response of 28 compounds + 1 control (miltefosine) in many different preclinical assays was carried out. These assays included a total of >50 different biological activity parameters [K_i_(nM), IC_50_(nM), Inhibiton (%), *etc*.)], 35 target proteins (P00374 dihydrofolate reductase, Q0GKD7 farnesyl pyrophosphate synthase, *etc*.), 28 cell lines (J774, HL-60, Jurkat, *etc*.), 40 assay organisms (*L. donovani*, *L. major*, *L. amazonensis*, *etc*.), and 2 microorganism development stages (amastigotes and promastigotes). In total, the outcome of the 29 compounds in 249 different pre-clinical assays was predicted.

#### 2.1.5 Data Sampling

In order to select the training and validation sets, we carry out the following steps. First, we ordered all assays according to the labels of output property c_0_ (IC_50_, K_i_, *etc*.) and the discrete values of the two main partitions, **c**_I_ and **c**_II._ After that, we assigned a value of set = train (t) or validation (v) from the beginning to the end following the pattern tttv. This allows us to use 75% (3/4) of the data for training the model and the remaining 25% (1/4) of the data for validation. The sorting of the cases by c_0_, **c**_I_, and **c**_II_ ensures a more representative distribution of all data strata or data subsets (properties, target proteins, pathogen species, etc.) in both training and validation series. The random sorting of all cases within each subset (same property label c_0_, or assay conditions c_I_ and c_II_) ensured a higher randomness of the sampling. In such a way, we have carried out one stratified, random, and representative data sampling. As result, we included a total of 6711 positive assays (f(v_ij_) = 1) and 102,678 negative or control assays (f(v_ij_) = 0) in training series. In addition, we included a total of 2050 positive assays (f(v_ij_) = 1) and 34,212 negative or control assays (f(v_ij_) = 0) in validation series.

### 2.2 Experimental Methods

#### 2.2.1 Synthesis of 2-(Hetero)aroylpyrroles **5**

**Scheme 1:**
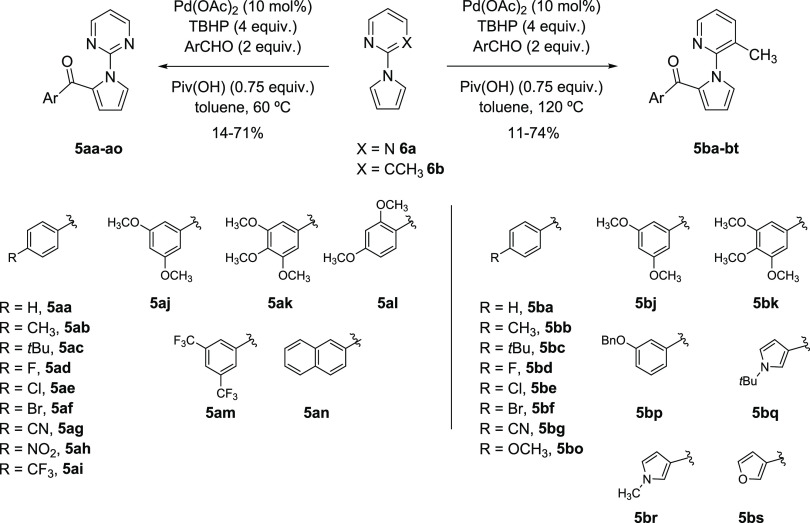
Synthesis of 2-Acylpyrroles **5a** and **5b** Screened against *L. amazonensis* and *L. donovani*

Acylpyrroles **5aa–an** and **5ba–bs** were synthesized following a procedure previously developed by us (Scheme 1).^15^ Acylation of 2-(1*H*-pyrrol-1-yl)pyrimidine **6a** and 3-methyl-2-(1*H*-pyrrol-1-yl)pyridine **6b** with the corresponding (hetero)aromatic aldehydes was carried out using Pd(OAc)_2_ as the catalyst, TBHP as the oxidant, and pivalic acid as the additive in dry toluene as the solvent. The reactions were carried out in sealed tubes at 60 °C (for **6a**) or 120 °C (for **6b**) for 1.5–7 h.

### 2.3 Details for Pre-clinical Assays

#### 2.3.1 Parasites and Culture Procedure

The following species of *Leishmania* were used: *L. donovani* (MHOM/IN/80/DD8) was purchased (ATCC, USA) and *L. amazonensis* (MHOM/Br/79/Maria) was kindly provided by Prof. Alfredo Toraño (Instituto de Salud Carlos III, Madrid). Promastigotes were cultured in Schneider’s insect medium supplemented with 10% heat-inactivated fetal bovine serum (FBS) and 1000 U/L of penicillin plus 100 mg/L of streptomycin in 25 mL culture flasks at 26 °C.

#### 2.3.2 *In Vitro* Promastigote Susceptibility Assay

The biological assay was carried out following previously published protocols.^27,28^ Concisely, promastigotes (2.5 × 10^5^ parasites/well) from the log phase have been cultured in 96-well plastic plates. New samples of the compounds have been prepared according to the protocol described above, and subsequently solutions of the chemical compound to be assayed have been dissolved in DMSO at 50 mg/mL. We performed serial dilutions 1:2 of the compounds in fresh culture medium (100, 50, 25, 12.5, 6.25, 3.12, 1.56, and 0.78 μg/mL) up to a 200 μL final volume. Growth control and signal-to-noise control were also included. The final solvent (DMSO) concentrations never exceeded 0.5% (v/v) warranting no effect on parasite proliferation or morphology. After 48 h at 26 °C, 20 μL of a 2.5 mM resazurin solution was added to each well and the plates were returned to the incubator for another 3 h. The relative fluorescence units (RFU) (535 nm–590 nm excitation–emission wavelength) was determined in a fluorimeter (Infinite 200Tecan i-Control). Growth inhibition (%) was calculated by 100 – [(RFU treated wells – RFU signal-to-noise)/(RFU untreated – RFU signal-to-noise) × 100]. All tests were carried out in triplicate. Miltefosine (Sigma-Merck, Madrid, Spain) was used as the reference drug and was evaluated under the same conditions. The efficacy of each compound was estimated by calculating the IC_50_ (concentration of the compound that produced a 50% reduction in parasites) using a multinomial probit analysis incorporated in SPSS software v21.0. The selectivity index (SI) was calculated as the ratio between cytotoxicity (CC_50_) and activity against parasites (IC_50_).

#### 2.3.3 *In Vitro* Intracellular Amastigote Susceptibility Assay

The assay was carried out as previously described.^29^ Briefly, 5 × 10^4^ J774 macrophages and stationary promastigotes in a 1:5 ratio were seeded in each well of a microtiter plate, suspended in 200 μL of culture medium, and incubated for 24 h at 33 °C in a 5% CO_2_ chamber. After this first incubation, the temperature was increased up to 37 °C for another 24 h. Thereafter, cells were washed several times in culture medium by centrifugation at 1.500*g* for 5 min in order to remove free non-internalized promastigotes. Finally, the supernatant was replaced by 200 μL/well of culture medium containing 2-fold serial dilutions of the test compounds as in promastigotes assay. Growth control and signal-to-noise were also included. Following incubation for 48 h at 37 °C and 5% CO_2_, the culture medium was replaced by 200 μL/well of the lysis solution (RPMI-1640 with 0.048% HEPES and 0.01% SDS) and incubated at room temperature for 20 min. Thereafter, the plates were centrifuged at 3.500*g* for 5 min and the lysis solution was replaced by 200 μL/well of Schneider’s insect medium. The culture plates were then incubated at 26 °C for another 4 days to allow transformation of viable amastigotes into promastigotes and proliferation. Afterward, 20 μL/well of 2.5 mM resazurin was added and incubated for another 3 h. Finally, fluorescence emission was measured and IC_50_ was estimated as described above. All tests were carried out in triplicate. Miltefosine (Sigma-Merck, Madrid, Spain) was used as the reference drug and was evaluated under the same conditions. The IC_50_ and SI were calculated as in the previous section.

#### 2.3.4 Cytotoxicity Assay on Macrophages

The assay was carried out as previously described.^30^ J774 macrophages cell lines were seeded (5 × 10^4^ cells/well) in 96-well flat-bottom microplates with 100 μL of RPMI 1640 medium. The cells were allowed to attach for 24 h at 37 °C and 5% CO_2_, and the medium was replaced by different concentrations of the compounds in 200 μL of medium and exposed for another 24 h. Growth controls and signal-to-noise were also included. Afterward, a volume of 20 μL of 2.5 mM resazurin solution was added, and plates were returned to the incubator for another 3 h to evaluate cell viability. The reduction of resazurin was determined by fluorometry as in the promastigote assay. Each concentration was assayed three times. The cytotoxicity effect of compounds was defined as the 50% reduction of cell viability of treated culture cells with respect to untreated culture (CC_50_) and was calculated using a multinomial probit analysis incorporated in SPSS software v21.0.

## 3 Results and Discussion

### 3.1 SOFT.PTML Model

As mentioned in the introduction, the IFPTML algorithm is useful for finding predictive models for multiobjective optimization of compounds. In fact, we have already used IFPTML models for the study of new pyrroloisoquinolines *vs* different *Leishmania* species.^16^ However, all steps of IFPTML analysis needed to be performed on different software and/or using different manual operations. Therefore, we decided to use our SOFT.PTML software for the development of IFPTML models for prediction of antileishmanial compounds.

**Figure 4:**
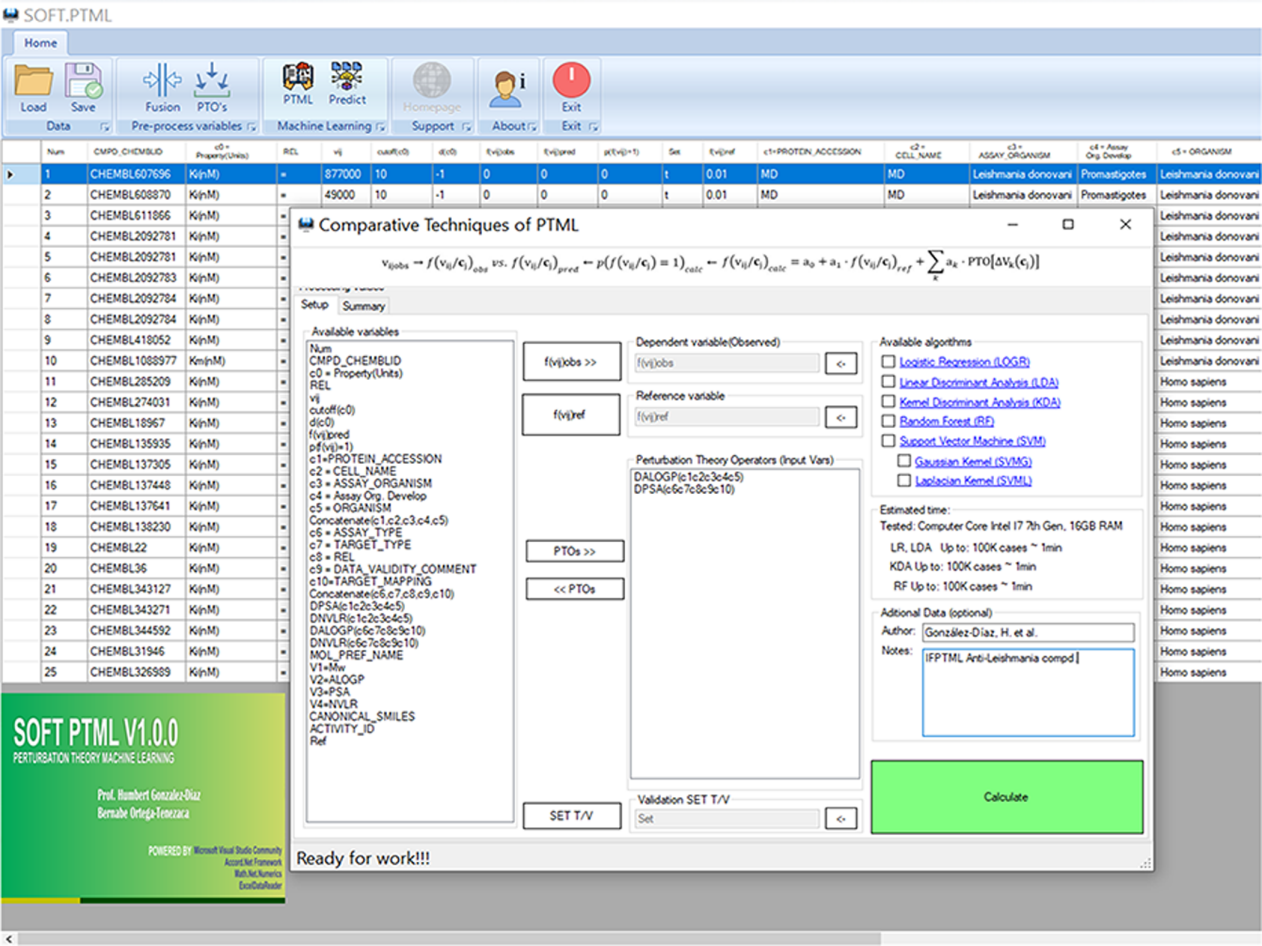
SOFT.PTML interface for analysis of ChEMBL antileishmanial compounds data.

The same dataset^16^ containing n = 109,389 preclinical assays was selected and re-processed with the SOFT.PTML software. Figure 4 shows the user-friendly interface of the software with the IF, PT, and ML stages integrated in a single application, which allowed exploring different ML techniques in a more automatic way. Specifically, logistic regression (LOGR), support vector machine (SVM), and random forests (RF) algorithms were studied.^31−33^Table 1 summarizes the results obtained with the different algorithms (see the Supporting Information 1 for details of the dataset used and detailed results of the model for each case).

**Table 1:** SOFT.PTML Analysis Results

						f(*v_ij_*)_obs_	
PTML model	ML	dataset	stat param	param value	set f(*v_ij_*)_pred_	0	1
1	LOGR	training	Sp	0.9885	0	101,493	1233
			Sn	0.9885	1	1185	5478
			Ac	0.9779	total		
		validation	Sp	0.9888	0	33,828	407
			Sn	0.8191	1	384	1843
			Ac	0.9783	total		
2	RF	training	Sp	0.9985	0	102,519	503
			Sn	0.9985	1	159	6208
			Ac	0.9939	total		
		validation	Sp	0.9934	0	33,985	315
			Sn	0.8600	1	227	1935
			Ac	0.9851	total		
3	SVM	training	Sp	0.9972	0	102,388	988
			Sn	0.9972	1	290	5723
			Ac	0.9883	total		
		validation	Sp	0.9944	0	34,021	757
			Sn	0.6636	1	191	1493
			Ac	0.974	total		

In this study, a total of 145,851 cases (pre-clinical assays outcomes) distributed in 109,389 cases in training series and 36,462 cases in validation series have been analyzed. The sensitivity (Sn) and specificity (Sp) values obtained from these algorithms were studied using the IFPTML strategy both in the training and external validation series of pre-clinical assays. It should be noted that all algorithms gave interesting results. However, the IFPTML-SVM model was discarded because it had a very low value of Sn = 0.6636 in validation series. The IFPTML-RF model was also discarded because although the results were very promising (Sp ≈ Sn = 0.8–0.98 range), the model itself is markedly more complex than the linear models. Therefore, the linear model IFPTML-LOGR was selected as the most appropriate based on the Ocam’s razor or parsimony principle.^34,35^ The equation of the IFPTML-LOGR model is the following (eq 2):
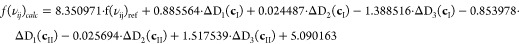
2
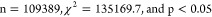


#### 3.1.1 All-in-One *vs* Multisoftware Strategy

SOFT.PTML uses an all-in-one strategy implementing all stages (IF, PT, and ML) of the IFPTML algorithm in the same platform. To validate the all-in-one strategy, it was necessary to demonstrate that this software was capable of reproducing the results obtained with the multisoftware strategy. Therefore, we decided to compare the IFPTML-LDA model with the multisoftware strategy (eq 3) with the IFPTML-LOGR model because both models are linear equations with the same form, despite using different ML techniques (LDA and LOGR).
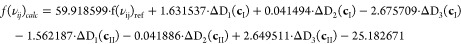
3
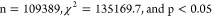


Both IFPTML models begin with a reference function f(*v_ij_*)_ref_, whose value represents the prior probability with which a compound selected at random can give a positive outcome f(v_ij_) = 1 of the specific parameter c_0_ under conditions c_j_. This function is used to specify within the equation the biological parameter (IC_50_, K_i_, MIC, etc.) to be studied. Next, the PTO_ki_(D_ki_, c_j_) = ΔD_ki_(c_j_) values are added to measure variations/perturbations. It should be highlighted that both IFPTML-LOGR and IFPTML-LDA models reached similar values of Sn and Sp in training and validation series. It was also found that the ratio between the coefficients (a_k_) of the PTO_ki_(D_ki_, c_j_) variables in both models is exactly constant = 2.0. This indicates that, except for a scale factor of 2, both equations give equal weight to the different variables and should give similar results. In fact, we found a correlation coefficient of R = 0.98 for the f(*v_ij_*)_calc_ values obtained with eq 2*vs*eq 3. This result demonstrates that the all-in-one strategy implemented in SOFT.PTML is capable of reproducing the results obtained with the multisoftware strategy, using a single program with a user-friendly interface, which makes the work notably easier and faster.

#### 3.1.2 Computational and Experimental Study of 2-Acylpyrroles

A case study is presented to illustrate the use of SOFT.PTML models for discovery of antileishmanial compounds in practice. As stated before, we focused on 2-acylpyrroles **5a** and **5b**, whose synthesis has been previously reported by us,^15^ because they combined structural features of related pyrrole derivatives^11−14^ with promising antileishmanial activity. To our knowledge, no previous studies on their antileishmanial activity have been reported. We describe herein the *in vitro* assays and in-depth computational screening of these compounds. First, the synthesis of new samples of 2-acylpyrroles **5a** and **5b** with pre-clinical assay quality was carried out. Next, these compounds were tested against two species of *Leishmania* in different development stages. These experimental studies included two biological activity parameters (IC_50_ and CC_50_) for two *Leishmania* species (*L. amazonensis* and *L. donovani*). Finally, the study was closed with a wide computational screening of these compounds *vs* many *Leishmania* species in different stages and multiple target proteins.

#### 3.1.3 Preparative Organic Synthesis

Our group has recently reported the synthesis of a variety of 2-(hetero)aroylpyrroles through a Pd(II)-catalyzed acylation of pyrrole with aldehydes^36^ in the presence of an oxidant, using 2-methylpyridinyl and 2-pyrimidyl as directing groups.^15^ This radical C–H activation reaction^37−41^ is a good catalytic alternative to classical acylation methods (Friedel–Crafts, Vilsmeier–Haack, or Houben–Hoesch type acylation reactions), which minimizes the production of waste as it does not require the use of stoichiometric amounts of Lewis or protic acids. Thus, we had demonstrated that the use of 2-pyrimidinyl directing group led to C-2 metalation of pyrrole using Pd(OAc)_2_ as the pre-catalyst in toluene, which were acylated with aldehydes in the presence of TBHP as the oxidant and pivalic acid as the additive. The procedure could be efficiently extended to a series of aldehydes with different substitution patterns on the aromatic ring, obtaining 2-acylpyrroles **5aa–an** (Scheme 1), though diacylation could not be completely avoided. However, under the same experimental conditions, the use of 3-methyl-2-pyridinyl directing group led to the formation of 2-acylpyrrole derivatives **5ba–bs** in moderate to good yields, except when electron-withdrawing substituents were present in the aromatic ring (Scheme 1).

#### 3.1.4 Antileishmanial Activity Pre-Clinical Assay

The 2-(hetero)aroylpyrrole derivatives **5a** and **5b** were tested against *L. amazonensis* and *L. donovani*, which are responsible for the two main clinical forms of this neglected tropical disease, cutaneous and visceral leishmaniasis, respectively (Table 2). We performed *in vitro* promastigote and *in vitro* intracellular amastigote susceptibility assays (IC_50_) and cytotoxicity assays (CC_50_) on the J774 cell line of macrophages using miltefosine as the drug of reference (see Materials and Methods), and the corresponding selectivity indexes (SI) were calculated. Detailed information of the biological activity of the more interesting compounds, including the compound code, concentration, repeated measures of biological activity, average values, can be found in the Supporting Information 2. This file also contains the graphic representations of dose–effect curves for these compounds and the drug of reference, miltefosine.

**Table 2:** IC_50_ Leishmanicidal and Cytotoxic Effects from 2-Acylpyrrole Series **5a** and **5b** (Expressed as μM) on *In Vitro* Promastigote Assays

		*L. amazonensis*		*L. donovani*		macrophages J774
entry	comp.	IC_50_ ± SD (μM)^a^	SI^b^	IC_50_ ± SD (μM)^a^	SI^b^	CC_50_ ± SD (μM)^c^
1	**5aa**	259.58 ± 40.72	>1.55	N/A^d^		401.17^e^
2	**5ab**	N/A^d^		N/A^d^		381.24^e^
3	**5ac**	32.88 ± 0.74	>2.77	58.54 ± 7.04	>1.55	91.02 ± 6.55
4	**5ad**	N/A^d^		N/A^d^		270.19 ± 26.30
5	**5ae**	117.09 ± 10.92	>3.01	189.77 ± 6.76	>1.86	352,46^e^
6	**5af**	67.18 ± 4.94	>4.54	118.18 ± 25.81	>2.58	304.72^e^
7	**5ag**	N/A^d^		N/A^d^		364.59^e^
8	**5ah**	N/A^d^		N/A^d^		339.82^e^
9	**5ai**	48.02 ± 1.61	>3.13	42.82 ± 0.61	>3.51	150.36 ± 49.40^e^
10	**5aj**	32.55 ± 0.64	>2.71	210.90 ± 32.77	>0.42	88.29 ± 3.36^e^
11	**5ak**	N/A^d^		N/A^d^		87.27 ± 7.37^e^
12	**5al**	N/A^d^		N/A^d^		224.28 ± 46.89^e^
13	**5am**	57.34 ± 0.25	>4.53	198.54 ± 25.36	>1.31	259.56^e^
14	**5an**	36.11 ± 1.23	>3.12	58.50 ± 3.34	>1.93	112.82 ± 39.19^e^
15	**5ba**	152.77 ± 21.65	>2.50	N/A^d^		381.23^e^
16	**5bb**	149.20 ± 3.84	>2.43	119.64 ± 14.98	>3.02	361.87^e^
17	**5bc**	30.87 ± 3.11	>10.17	221.31 ± 36.90	>1.42	314.05^e^
18	**5bd**	16.87 ± 0.73	>10.67	7.78 ± 0.27	>23.15	136.96 ± 36.42
19	**5be**	51.46 ± 4.72	>2.89	20.69 ± 0.98	>7.19	148.74 ± 19.33
20	**5bf**	38.10 ± 0.85	>3.14	19.87 ± 1.47	>6.01	119.51 ± 37.36^e^
21	**5bg**	191.77 ± 12.04	>1.81	315.43 ± 60.80	>1.10	348.04^e^
22	**5bj**	71.06 ± 7.81	>1.53	43.51 ± 0.59	>0.80	108.45 ± 5.76^e^
23	**5bk**	N/A^d^		N/A^d^		283.78
24	**5bo**	224.26 ± 14.71	>1.53	172.92 ± 60.34	>1.98	342.07^e^
25	**5bp**	207.96 ± 23.62	>1.31	184.22 ± 8.67	>1.47	356.76^e^
26	**5bq**	209.16 ± 4.69	>1.56	92.81 ± 7.68	>1.08	325.31^e^
27	**5br**	226.54 ± 13.42	>1.66	N/A^d^		376.90^e^
28	**5bs**	N/A^d^		N/A^d^		396.40^e^
29	miltefosine	30.67 ± 8.80	1.80	0.24 ± 0.02	230.83	55.40 ± 4.19

a IC_50_: concentration of the compound that produced a 50% reduction in parasites; SD: standard deviation.

b SI: selectivity index, SI = CC_50_/IC_50_.

c CC_50_: concentration of the compound that produced a 50% reduction of cell viability in treated culture cells with respect to untreated ones.

d N/A: not active at the maximum dose tested (100 μg/mL).

e CC_50_ values, expressed as μM, correspond to 100 μg/mL, which was the higher doses tested.

The performance of each *N*-pyrimidin-2-yl acylated pyrrole **5a** was compared with that of the corresponding *N*-(3-methylpyridin-2-yl) derivative **5b** (Table 2). The bioactivities of some compounds of both series compare well in terms of activity and selectivity against *L. amazonensis* promastigotes*.* The aromatic substitution pattern of the acyl group plays an important role in the antileishmanial activity of these pyrrole derivatives. In some cases, we observed similar trends in the bioactivity profile for pyrimidine derivatives **5a** and the corresponding pyridines **5b**. For example, the 4-^*t*^butylphenyl pyrrolyl methanones **5ac**/**5bc** and the 3,5-disubstituted phenyl pyrrolyl methanones **5aj**/**5bj**, with electron-donating (MeO) substituents, showed IC_50_ in a similar micromolar range to miltefosine (Table 2, entries 3 *vs* 17 and 10 *vs* 22). The parallel behavior was maintained also for trisubstituted derivatives **5ak**/**5bk**, which were both inactive under our bioassay conditions (Table 2, entries 11 *vs* 23). However, there were significant differences in the 2-(hetero)aroylpyrroles derivatives with halogenated aromatic rings. In particular, in the pyridine series, **5bd** (R = F) was found to be more active and selective than the drug of reference (miltefosine) (IC_50_ = 16.87 ± 0.73 μM, SI > 10.67), while the corresponding pyrimidine derivative **5ad** (R = F) was inactive (Table 2, entry 17 *vs* entry 4). It also should be pointed out that compound **5an**, where the phenyl ring had been changed to a naphthyl ring, showed similar activity to the drug of reference with better selectivity (Table 2, entry 14).

The same set of 2-(hetero)aroylpyrroles **5a,b** was also tested on promastigotes forms of *L. donovani* (Table 2). All compounds were considerably less active and selective than miltefosine. Halogenated pyridine derivatives **5bd**–**5bf** presented the best profiles, **5bd** again being the most active and selective of all 2-acylpyrroles (IC_50_ = 7.78 ± 0.27 μM and SI > 23.15). However, it should be highlighted that all tested pyrrole derivatives were less toxic than miltefosine with values of concentration of the compound that produces 50% reduction of cell viability (cytotoxic concentration, CC_50_) in the range 87–401 μM in J774 cells. This is a promising result, taking into account high toxicity (low selectivity) of marketed available drugs.^3^

Then, one compound of each series was further tested *in vitro* on *L. amazonensis* and *L. donovani* amastigotes (Table 3). Pyrimidine derivative **5bc** showed good performance with an activity similar to miltefosine and better selectivity (IC_50_ = 60.55 ± 7.88 μM, SI > 5.19) against *L. amazonensis*. Nevertheless, pyridine derivative **5bc** presented bad results in terms of activity and selectivity (IC_50_ = 153.27 ± 9.11 μM, SI > 1.99).

**Table 3:** IC_50_ Leishmanicidal and Cytotoxic Effects from 2-Acylpyrroles **5af** and **5bc** (Expressed as μM) on *in Vitro* Amastigote Assay

		*L. amazonensis*		*L. donovani*		macrophages J774
entry	compound	IC_50_ ± SD (μM)^a^	SI^b^	IC_50_ ± SD (μM)^a^	SI^b^	CC_50_ ± SD (μM)^c^
1	**5af**	153.27 ± 9.11	>1.99	210.87 ± 30.26	>1.45	304.72^e^
2	**5bc**	60.55 ± 7.88	>5.19	N/A^d^		314.05^e^
3	miltefosine	47.55 ± 7.04	2.85	0.44 ± 0.05	307.70	135.93 ± 10.19

a IC_50_: concentration of the compound that produced a 50% reduction in parasites; SD: standard deviation.

b SI: selectivity index, SI = CC_50_/IC_50_.

c CC_50_: concentration of the compound that produced a 50% reduction of cell viability in treated culture cells with respect to untreated ones.

d N/A: not active at the maximum dose tested (100 μg/mL).

e CC_50_ values, expressed as μM, correspond to 100 μg/mL, which was the higher doses tested.

#### 3.1.5 IFPTML-Based Computational Screening of New Compounds

For this predictive study, we selected 28 compounds previously synthesized by our group (see structures on Scheme 1**)**, whose *in vitro* biological activity (IC_50_ values) *vs* two *Leishmania* species (*L. donovani*^42^ and *L. amazonensis*(43)) and cytotoxicity *vs* one cell line (J774 line of BALB/c mice macrophages^44^) has been carried out (Tables 2 and 3). However, there are >20 clinically relevant *Leishmania* species, such as *L. major*,^45^*L. mexicana*,^46^*L. aethiopica*,^47^*L. braziliensis*, *L. amazonensis*, *L. donovani*,^48^*L. infantum*,^49^*etc*. Therefore, it could be very interesting to know (a) other parameters (K_i_, K_m_, etc.) of *in vitro* biological activity *vs* specific target proteins and (b) the cytotoxicity of these compounds *vs* other human and animal cell lines such as Jurkat,^50^ Vero,^51^ THP-1,^52^HEK293,^53^ HeLa,^54^ HL-60,^55^ Sf9,^56^*etc*. Consequently, we decided to use our multioutput IFPTML model to perform an in-depth computational screening of the biological activity of these compounds in all the biological assays space. Thus, we ran a computational screening experiment involving calculation of 20,704 activity scores for 29 compounds (28 compounds + miltefosine as reference) in 647 different preclinical assays. These 647 preclinical assays of reference present unique combinations of the biological assay conditions c_0_ = parameter (K_i_, IC_50_, K_m_, *etc*.), c_1_ = target protein, c_2_ = cell line (J774, HeLa, HL60, *etc*.), c_3_ = organism (*L. major*, *L. mexicana*, *etc*.), or c_4_ = organism stage of development.

The following steps were performed. First, DRAGON software^25^ was used to calculate the entries of the vector of molecular descriptors for each compound. Next, the values of the molecular descriptors D_ki_ were substituted into the model, obtaining as output the scores of biological activity f(*v_ij_*)_calc_ for the *i*th compound in the *j*th assay. Finally, the scores of biological activity f(*v_ij_*)_calc_ were expressed in terms of relative deviation Δf(v_ij_)%_calc_ = 100·[f(*v_ij_*)_calc_ – f(v_mtfj_)_calc_]/f(v_mtfj_)_calc_. These relative scores express the deviation of the *i*th query compound from the reference, miltefosine (mtf). The predictions show, in general, a higher relative biological activity score Δf(v_ij_)%_calc_ for the compound series **5b** than for series **5a** compared to the drug of reference (miltefosine). Specifically, compounds **5bc** and **5bp** show 1–4 fold higher relative biological activity score values compared to miltefosine. The prediction is consistent with our experimental findings for IC_50_ activity assays *vs L. amazonensis* and *L. donovani* and selectivity index in the J774 cell line (Table 4).

**Table 4:** Relative Biological Activity Score with Respect to Miltefosine

assay conditions c_j_^a^				relative variation in biological activity score^b^						
c_0_	c_1_	c_2_	c_3_	Δf(v_ij_)%_calc_ values						
property (units)	target protein	cell line	assay org.	**5am**	**5af**	**5bc**	**5bd**	**5bp**	n_j_	f(*v_ij_*)_ref_
Top species predicted with model										
IC_50_ (nM)	MD	MD	*L. amazonensis*	1.70	–0.06	2.09	0.30	2.02	724	0
IC_50_ (nM)	MD	THP-1	*H. sapiens*	3.17	–0.12	3.89	0.56	3.77	546	0
Inh. (%)	MD	MD	*L. donovani*	3.17	–0.12	3.89	0.56	3.77	2654	0.01
IC_50_ (u)	MD	MD	*L. aethiopica*	3.13	–0.12	3.84	0.56	3.73	81	0.82
EC_50_ (nM)	MD	MD	*L. major*	3.13	–0.11	3.84	0.56	3.72	482	0.01
GI (%)	MD	MD	*L. tropica*	3.00	–0.11	3.68	0.53	3.57	279	0.51
IC_50_ (nM)	MD	MD	*L. chagasi*	2.79	–0.10	3.42	0.50	3.31	85	0
										
Top cell lines predicted with model										
ratio	MD	J774.A1	*MD*	1.32	–0.05	1.62	0.23	1.57	84	0.91
SI	MD	L6	*MD*	3.39	–0.12	4.16	0.60	4.03	181	0.89
IC_50_ (u)	MD	J774.A1	*L. donovani*	3.38	–0.12	4.15	0.60	4.02	144	0.82
SI	MD	J774	*MD*	3.23	–0.12	3.97	0.58	3.85	65	0.91
IC_50_ (nM)	MD	THP-1	*H. sapiens*	3.15	–0.12	3.87	0.56	3.75	637	0
SI	MD	LLC-MK2	*MD*	3.02	–0.11	3.71	0.54	3.60	61	0.91
SI	MD	Vero	*MD*	2.98	–0.11	3.65	0.53	3.54	51	0.91
SI	MD	HepG2	*MD*	1.41	–0.05	1.72	0.25	1.67	63	0.91
IC_50_ (u)	MD	J774.A1	*L. donovani*	3.38	–0.12	4.15	0.60	4.02	144	0.82
Top target proteins predicted with model										
K_i_ (nM)	P07382	MD	*L. major*	3.75	–0.14	4.60	0.67	4.46	48	0.01
IC_50_ (nM)	O61059	MD	*L. mexicana*	3.29	–0.12	4.03	0.59	3.91	372	0
IC_50_ (nM)	P11166	MD	*L. mexicana*	3.27	–0.12	4.01	0.58	3.89	13,658	0
IC_50_ (nM)	O97467	MD	*L. mexicana*	3.27	–0.12	4.01	0.58	3.89	13,642	0
IC_50_ (nM)	Q0GKD7	MD	*L. major*	2.95	–0.11	3.62	0.53	3.51	76	0
Inh. (%)	P39050	MD	*L. donovani*	2.81	–0.10	3.44	0.50	3.34	53	0.01
Act. (%)	Q27686	MD	*L. mexicana*	2.36	–0.09	2.90	0.42	2.81	62	0.1
K_i_ (nM)	Q01782	MD	*L. major*	1.52	–0.06	1.86	0.27	1.81	46	0.01
Inh. (%)	E9BF75	MD	*L. donovani*	1.32	–0.05	1.62	0.24	1.58	68,577	0.01

a Act.(%) = activity (%), Inh. (%) = inhibition (%), GI (%) = growth inhibition (%), Cyt. (%) = cytotoxicity (%),SI = selectivity index = ratio CC_50_/IC_50_, IC_50_ (u) = IC_50_ (ug mL^–1^).

b Δf(v_ij_)%_calc_ = 100·[f(*v_ij_*)_calc_ – f(v_mtfj_)_calc_]/f(v_mtfj_)_calc_, f(*v_ij_*)_calc_ = calculated biological activity score of *i*th compound, f(v_mtfj_)_calc_ = calculated biological activity score of drug of reference miltefosine, MD = missing data.

Table 4 summarizes the results of scores of biological activity f(*v_ij_*)_calc_ calculated for some of the 28 compounds (**5am**, **5af**, **5bc**, **5bd**, and **5bp**), considering the most relevant organisms, cell lines, and target proteins. Compound **5bd** was predicted with a positive value Δf(v_ij_)%_calc_**=** 0.30 for IC_50_*vs L. amazonensis* promastigotes. It means that the model predicts this compound with high probability to be in the same range of activity than miltefosine. This result is in agreement with the experimental findings reported in the previous section, IC_50_ = 16.87 μM for compound **5bd** and IC_50_ = 30.67 for miltefosine *vs L. amazonensis* promastigotes. Compound **5bc** is also predicted with a positive value of Δf(v_ij_)%_calc_**=** 2.09 for IC_50_*vs L. amazonensis* promastigotes, which also matches with our experimental finding (IC_50_ = 30.87 μM for compound **5bc***vs* IC_50_ = 30.67 for miltefosine in *L. amazonensis* promastigotes assay). The same trend has been observed for other compounds (e.g., **5am**) that were also predicted with positive Δf(v_ij_)%_calc_ values approximately in the same range as miltefosine. Interestingly, compound **5af** is predicted with values Δf(v_ij_)%_calc_ lower than miltefosine (negative values of Δf(v_ij_)%_calc_) both *vs L. amazonensis* and *L. donovani* promastigotes, as observed in the experimental results (see Table 2).

In addition, the scores of biological activity of these compounds *vs* different cell lines were predicted. For this series, the cutoff for the scores of biological activity was 1.62 and 50 for n_j_. Only one assay per cell line has been shown. First, we focused on the selectivity index (SI = ratio CC_50_/IC_50_) of the compounds *vs* J774 cell lines because they were the experimental lines used. Specifically, compound **5bc** has a value of Δf(v_ij_)%_calc_ = 1.62 for cell line J774.A1, which means that this compound is expected to show a similar-to-higher probability than miltefosine of presenting positive SI, in agreement with our experimental results. In fact, compound **5bc** was found to have a SI >10.17, which is approximately 6 times the value for miltefosine with SI = 1.80. The model was able to reproduce, in general, the trends on SI values for all the compounds of both series for cell lines J774 and/or J774.A1. Similar results of positive SI were predicted with the IFPTML model for other cell lines not experimentally tested here. The higher values were calculated for cell lines: L6, J774.A1, J774 THP-1, LLC-MK2, Vero, and HepG2. This points to these lines as interesting targets for further testing the safety of these compounds in the future.

Finally, those proteins with the higher increase in biological activity score from the reference miltefosine were selected. In addition, proteins were filtered by the number of assays (n_j_) reported in the ChEMBL dataset for each protein. The proteins with higher n_j_ are the most studied and probably the most relevant due to the increased attention they are receiving. To select the cases that include the most relevant proteins, the cutoff for the scores of biological activity was Δf(v_ij_)%_calc_ = 1.62 and the cutoff for n_j_ = 45. Only one assay per protein is shown. According to the results obtained with the IFPTML model, the most plausible target proteins are the following: vifunctional dihydrofolate reductase-thymidylate synthase (P07382),^57^ glucose transporter (O61059),^58^ solute carrier family 2 facilitated glucose transporter member 1 (P11166),^59^ hexose transporter 1 (Q0GKD7),^60^ farnesyl pyrophosphate synthase (O97467),^61^ trypanothione reductase (P39050),^62^ pyruvate kinase (Q27686),^63^ pteridine reductase 1 (Q01782),^64^ and methionine-tRNA ligase (E9BF75).^65^ After a detailed inspection of all compounds active *vs* these proteins in our ChEMBL database, no significant similarity was found between the previously reported compounds and the 2-acylpyrrole derivatives tested here. Consequently, 2-acylpyrrole derivatives could be considered a new class of antileishmanial lead compounds that deserve further investigation. The detailed results of the computational study are released in Supporting Information 3.

## 4 Conclusions

We have shown that SOFT.PTML is a useful tool for developing predictive models for drug discovery. The software implements IFPTML algorithms in a user-friendly interface without the need to rely on multiple software to run the different stages (IF, PT, and ML) of the algorithm. More importantly, SOFT.PTML can process complex datasets with big data features (high volume, multiple outputs, multiple target proteins, cell lines, pathogen species, missing data, *etc*.). Specifically, the use of this software has been illustrated by processing a very large ChEMBL dataset (>145,000 cases) from preclinical assays against different *Leishmania* species. Among the different ML algorithms (SVM, LOGR, and RF) explored, the best model was IFPTML-LOGR, which estimates the probability with which multiple parameters (IC_50_, CC_50_, SI, *etc*.) of a new compound get to a desired level in pre-clinical assays with high specificity and sensitivity (80–98%) in both training and validation series. This result demonstrates that the all-in-one strategy implemented in SOFT.PTML is capable of reproducing the results obtained from the multisoftware strategy, using a single program with a user-friendly interface that makes the work noticeably easier and faster. The pre-clinical assays studied involve different *Leishmania* species and cell lines, as well as multiple target proteins. The use of the new tool has been illustrated in a practical case study, the 2-acylpyrrole derivatives. The *in vitro* evaluation of the leishmanicidal activity of 2-acylpyrrole series **5a** and **5b** against visceral (*L. donovani*) and cutaneous (*L. amazonensis*) leishmaniasis revealed that all tested 2-acylpyrroles showed very low cytotoxicity, CC_50_ > 100 μg/mL in J774 cells (highest tested dose). This is an important feature as drug toxicity is one of the main limitations of current chemotherapy for leishmaniasis. In particular, **5bd** (IC_50_ = 16.87 μM, SI > 10.67) was approximately 6-fold more potent and selective than the drug of reference (miltefosine) in *L. amazonensis* promastigote assays. These results point to 2-acylpyrroles as a new class of lead compounds worthy of further optimization as antileishmanial hits.

## Supporting Information Available

ChEMBL dataset used to train and validate the model, compounds codes, SMILE codes, preclinical assay conditions, observed values, predicted classifications, probabilities, *etc*. (XLSX)
Detailed information of the biological activity of the more interesting compounds, including the compound code, concentration, repeated measures of biological activity, average values; graphic representations of dose–effect curves for these compounds and the drug of reference, miltefosine. (XLSX)
Results of the computational study of the new series of 2-acylpyrrole derivatives (XLSX).

## Notes

The Supporting Information including all the data used in this paper is available free of charge on the ACS Publications website at DOI: 10.1021/acs.jcim.xxxxxx. The software SOFT.PTML used in this paper is also available upon request to corresponding author at humberto.gonzalezdiaz@ehu.eus. Not proprietary data is reported on this work. Materials and Methods describe all the theory at a level that allows a person skilled in the art could implement the method.
